# Efficacy of electrical cranial stimulation for treatment of psychiatric symptoms in patients with anxiety: A systematic review and meta-analysis

**DOI:** 10.3389/fpsyt.2023.1157473

**Published:** 2023-04-06

**Authors:** Feng-Chin Chung, Cheuk-Kwan Sun, Yi Chen, Yu-Shian Cheng, Weilun Chung, Ruu-Fen Tzang, Hsien-Jane Chiu, Ming-Yu Wang, Ying-Chih Cheng, Kuo-Chuan Hung

**Affiliations:** ^1^Department of Psychiatry, Tsyr-Huey Mental Hospital, Kaohsiung Jen-Ai's Home, Kaohsiung, Taiwan; ^2^Department of Emergency Medicine, E-Da Hospital, I-Shou University, Kaohsiung City, Taiwan; ^3^College of Medicine, School of Medicine for International Students, I-Shou University, Kaohsiung, Taiwan; ^4^Department of Psychiatry, Mackay Memorial Hospital, Taipei, Taiwan; ^5^Taoyuan Psychiatric Center, Ministry of Health and Welfare, Taoyuan, Taiwan; ^6^Institute of Hospital and Health Care Administration, National Yang-Ming Chiao Tung University, Taipei, Taiwan; ^7^Department of Psychiatry, China Medical University Hsinchu Hospital, China Medical University, Hsinchu, Taiwan; ^8^Department of Health Services Administration, China Medical University, Taichung, Taiwan; ^9^College of Public Health, National Taiwan University, Institute of Epidemiology and Preventive Medicine, Taipei, Taiwan; ^10^Research Center of Big Data and Meta-Analysis, Wan Fang Hospital, Taipei Medical University, Taipei, Taiwan; ^11^College of Medicine, School of Medicine, National Sun Yat-sen University, Kaohsiung, Taiwan; ^12^Department of Anesthesiology, Chi Mei Medical Center, Tainan, Taiwan

**Keywords:** cranial electrotherapy stimulation, anxiety, meta-analysis, depression, insomnia

## Abstract

**Background:**

Therapeutic effects of electrical cranial stimulation (CES) in patients suffering from anxiety remained unclear. This meta-analysis aimed at investigating acceptability and therapeutic efficacy of CES against anxiety, depression, and insomnia for patients who experienced symptoms of anxiety.

**Methods:**

Major electronic databases were searched from inception until December 10, 2022 for randomized controlled trials (RCT) focusing on therapeutic effectiveness of CES in patients whose primary complaints included anxiety. Effect sizes (ES) for different treatment outcomes were estimated by using generic inverse variance method.

**Results:**

Eight RCTs were identified including a total of 337 participants. The therapeutic effectiveness of CES was significantly better than that in the control groups for anxiety (ES=-0.96, *p* <0.00001, eight trials, 337 patients), depression (ES=-0.69, *p*=0.003, five trials), and insomnia (ES=-1.02, *p* = 0.0006, three trials) in those who presented with symptoms of anxiety. Subgroup analyses found that CES was equally effective regardless of comorbid presentation of depressive symptoms (ES=-0.94 in patients with anxiety only vs. ES=-1.06 in those with depression and anxiety) and whether CES was used as monotherapy or add-on therapy to medications (ES = −0.88 vs. ES = −1.12, respectively). Moreover, subgroup analysis of RCTs using the same device “Alpha-Stim” for CES was more effective in alleviating anxiety than sham controls (ES = −0.88, *p* < 0.00001, four trials, 230 patients). Regarding acceptability, the use of CES did not increase the risk of treatment-related dropout compared to the control group (RR = 1.26, *p* = 0.57, I^2^ = 0%, four trials, 324 patients).

**Conclusion:**

Our study supported the use of CES for symptoms of anxiety, depression, and insomnia in those suffering from anxiety with fair acceptability and demonstrated the efficacy of “Alpha-Stim”, the most commonly used device for CES, in this patient population.

**Systematic review registration:**

https://www.crd.york.ac.uk/prospero/, identifier: CRD42022382619.

## 1. Introduction

Anxiety disorders, the most commonly diagnosed psychiatric disorders with an estimated prevalence between 4.8% and 10.9% worldwide (1), are often comorbid with other psychiatric problems such as depression and insomnia (2). Although pharmacological and psychosocial interventions are well accepted treatment strategies (1), there are uncertainties about the long-term safety and side effects of pharmacological treatments (3). In addition, two of the reported downsides of psychosocial interventions included failure to achieve a full response in a significant portion of patients suffering from anxiety (4) as well as a high dropout rate (5). Therefore, alternative treatments are frequently sought in patients with anxiety.

Neuro-stimulation, a potential treatment alternative, has been increasingly used for anxiety disorders and a variety of psychiatric disorders (6). There are different methods of delivering such stimulation to the brain. One such approach, commonly referred to as “transcranial electrical stimulation,” targets the brain more directly with devices being placed in close proximity to the brain (i.e., scalp or forehead) (e.g., repetitive transcranial magnetic stimulation and transcranial direct current stimulation) (7). Another method such as cranial electrical stimulation (CES) delivers weaker stimulation to the brain with electrodes being placed farther from the brain (i.e., eyelids or ears) (8). In addition, another system known as “transcutaneous electrical acupoint stimulation” (TEAS), which combines traditional Chinese acupuncture with neuro-stimulation, has been used for relieving anxiety during invasive medical procedures (i.e. *in vitro* fertilization) (9) and more recently for treating post-traumatic stress disorder (10). Among these approaches, CES has been approved by the U.S. Food and Drug Administration (FDA) and accepted as a therapeutic alternative for anxiety, depression, insomnia, and various pain conditions. Nevertheless, despite being approved by FDA for more than four decades (8), the efficacy of CES against psychiatric disorders remained unclear mainly due to the poor quality of evidence (11).

Although the exact therapeutic mechanism of CES is still unknown (8), previous investigations have suggested that CES may have direct neuromodulation effects on brain regions involving emotional controls including the limbic system, reticular activating system, and the hypothalamus (12), thereby enhancing parasympathetic but suppressing sympathetic tones of the autonomic nerve system (8, 13). Another study also found that CES treatment was linked to an increase in alpha frequencies which is associated with relaxation (14). However, despite a number of clinical trials endorsing the therapeutic effects of CES against anxiety (15–17), a recent review concluded that the evidence of its efficacy was still insufficient because most studies had a high risk of bias attributed to inadequate blinding and small sample sizes (11). Moreover, notwithstanding the report of CES efficacy against anxiety in two previous meta-analyses (18, 19), both studies involved some methodological issues. While one was dated in 1995 with the inclusion of randomized controlled trials (RCTs) that had small sample sizes and utilized devices (e.g., Neurotone 101) that is no longer available (18), the other more recent one showed a significant level of heterogeneity despite the inclusion of newer trials (19). Furthermore, both meta-analyses included a mixed group of participants diagnosed with a variety of psychiatric or medical conditions, which may contribute to heterogeneity of their results (18, 19). Another potential confounding factor that may obscure the significance of outcome was heterogeneity arising from therapeutic strategies. For instance, a previous meta-analysis included RCTs in which some used CES as monotherapy while the others adopted it as an add-on treatment to medications (19). Therefore, the efficacy of CES as a monotherapy against anxiety remained unclear.

Therefore, to focus on the therapeutic effects of CES in patients with anxiety, the present meta-analysis only selected RCTs that recruited patients whose primary complaints included symptoms of anxiety. We also investigated the therapeutic efficacies of CES for symptoms of depression and insomnia in this population and further conducted subgroup analyses to elucidate the efficacies of CES as a component in different therapeutic strategies (monotherapy vs. add-on treatment).

## 2. Methods

### 2.1. Protocol registration

The current meta-analysis, which was conducted in compliance with the Preferred Reporting Items for Systematic Reviews and Meta-Analyses (PRISMA) guidelines (20), was registered in PROSPERO (CRD42022382619).

### 2.2. Search strategy and data sources

We used pre-defined strategies to search five major databases, namely the Embase, Cochrane Library, Web of science, ScienceDirect and Medline from inception until February 27, 2023 to identify RCTs that assessed the efficacy of CES against anxiety without ethnicity and language restrictions. The search terms applied for the screening of eligible studies included “cranial electrotherapy stimulation” AND “anxiety” with the use of a combination of free-text terms and controlled vocabulary (e.g., MeSH) to facilitate literature search. The search strategy for one of the databases (i.e., Medline) is provided in Supplementary Table 1. Besides, the reference lists of all eligible RCTs and retrieved review articles were screened for additional relevant studies.

### 2.3. Study selection and data extraction

After identification of potentially eligible records, the titles and abstracts were initially screened by two independent authors based on the PICO (i.e., population, intervention, comparator, and outcomes) criteria:(1) Population: We included studies that clearly indicated “symptoms of anxiety” in their participants as well as those with anxiety-related diagnostic criteria (e.g., mixed anxiety-depression) according to that defined in individual studies regardless of the presence of other psychiatric comorbidities. The studies were then independently examined by two reviewers to determine eligibility of the participants with disagreements being resolved through discussion; (2) Intervention: use of CES as monotherapy or combination therapy with medications; (3) Comparator: use of sham stimulation, no treatment, or interventions other than CES as a control group; and (4) Outcomes: improvement in anxiety symptoms. Only RCTs were included for analysis; therefore, studies published in the following formats were excluded: letters, meta-analyses, conference abstracts, reviews, and case reports. In addition, we did not include RCTs that assessed the therapeutic use of other forms of brain stimulation (e.g., repetitive transcranial magnetic stimulation). Any disagreement was solved through discussion with a third author.

The following data were extracted independently by two authors: publication-specific details (e.g., journal, first-author name, and publication year) and study characteristics (e.g., number of patients, country, and follow-up duration).

### 2.4. Primary and secondary outcomes

The primary outcome was change in anxiety intensity using validated assessment methods such as the State Anxiety Inventory and Profile of Mood States. The diagnosis of anxiety was in accordance with that of each study. “Focusing on the primary outcome, subgroup analyses based on the period of publication (i.e., 1970–1990 vs. 2007–2021), the type of CES (e.g., Alpha-Stim), anxiety with or without depressive symptoms, and the use of CSE as monotherapy or combination therapy were performed. Secondary outcomes included improvements in depressive symptoms, insomnia and acceptability of CES based on the number of participants who discontinued treatment in each group.

### 2.5. Quality assessment and certainty of evidence

Cochrane's “risk of bias” assessment tool was used to determine the quality of the included studies. Following the evaluation of sequence generation, allocation concealment, blinding of assessors, blinding of participants, incomplete outcome data, selective outcome reporting, as well as the presence of other biases, each study was allocated to the category of low/unclear/high risk of bias. Based on the Grading of Recommendations Assessment, Development and Evaluation (GRADE) framework (26), the certainty of evidence for the outcomes of interest was investigated. A discussion involving two independent authors was held to resolve any disagreements regarding the overall certainty of the evidence. Disagreements were settled through the involvement of a third author.

### 2.6. Statistical analysis

All effect sizes are presented as risk ratios (RRs) or standardized mean difference (SMD) with 95% confidence interval (CI). The events/total number of participants or SMD (SE) were entered into the Review Manager 5 (RevMan 5.4; Copenhagen: The Nordic Cochrane Center, The Cochrane Collaboration, 2014) using the Mantel-Haenszel (MH) method or generic inverse-variance method where appropriate. We judged the effect sizes as minimal (SMD<0.2), small (SMD: 0.2–0.5), medium (SMD: 0.5–0.8), and large (SMD>0.8) as previously reported (27). Heterogeneity among studies was reported using the I^2^ statistics (low: ≤50%; moderate: 50% to 75%, high: ≥75%) (28). The reliability of the available outcomes was assessed using the leave-one-out sensitivity analysis. We examined the potential publication bias by visual inspection of a funnel plot. A *p* < 0.05 was considered statistically significant in all outcomes.

## 3. Results

### 3.1. Study selection and characteristics

**Figure 1:**
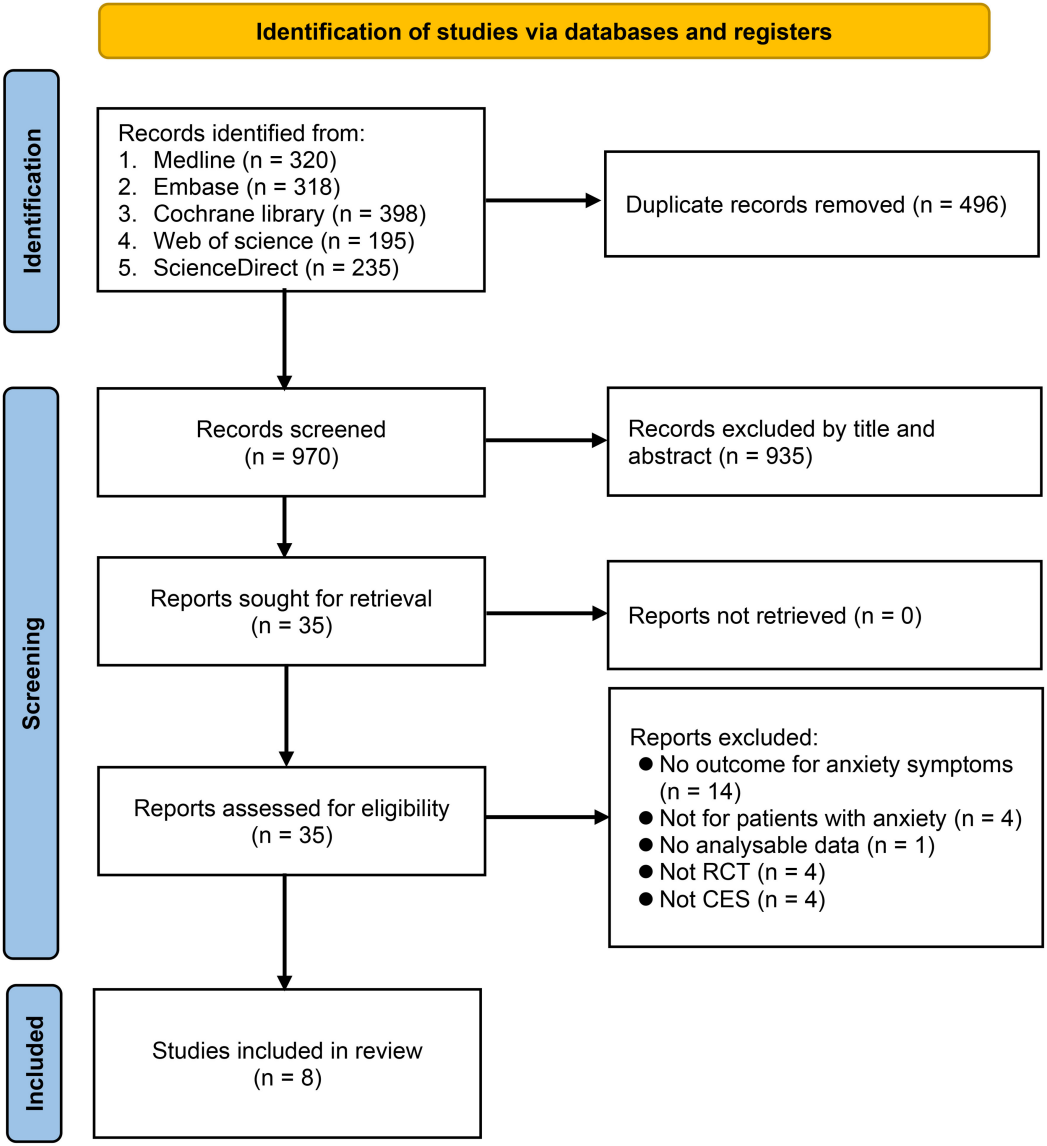
Flowchart for study selection. CES, cranial electrical stimulation; RCT, randomized controlled trial.

Of the 1,466 articles identified through comprehensive literature search, 496 duplicates and 935 that failed to meet our inclusion criteria after title and abstract screening were excluded. As a result, 35 articles were eligible for full-text review. We then excluded 27 studies because they did not discuss anxiety symptoms (*n* = 14), included patients without anxiety (*n* = 4), did not provide meaningful data (*n* = 1), did not use CES (*n* = 4), or were not RCTs (*n* = 4) (Supplementary Table 2). Finally, eight studies published between 1972 and 2021 were included in the present meta-analysis (15–17, 21–25). A summary of the study selection process is shown in Figure 1.

Table 1 summarizes the characteristics of the eight included trials. Seven studies investigated the therapeutic efficacy of CES in adults (mean age: 25.6–43.1 years), whereas one study focused on adolescents (mean age: 11–12 years) (16). Seven trials were conducted using a randomized controlled design, while one study adopted both randomized controlled and cross-over designs (24). The proportion of females ranged from 16.7% to 100% with three studies primarily focusing on female participants (range: 78.9%−100%) (23–25). The sample sizes ranged from 10 to 120. In the intervention group, four studies used Alpha-Stim (15–17, 22), while the other four trials used different types of devices (21, 23–25). Seven trials applied sham stimulation in their control groups, whereas one did not provide information about the use of sham devices in comparison groups (17). Seven studies reported the treatment duration (range: 1–6 weeks) and number of sessions (range: 5–42), while one study did not provide relevant details (22). Regarding the use of medications, three studies prohibited medication use (16, 21, 22), two trials used CES as an add-on therapeutic strategy to medications (17, 24), and three studies allowed the use of medications (15, 23, 25). The included studies were conducted in three countries, namely Korea (*n* = 1), China (*n* = 2), and USA (*n* = 5).

**Table 1:** Summary of characteristics of studies in the current meta-analysis.

**References**	**Diagnosis (Criteria)**	**Design**	**Comparison**	**N**	**Duration (weeks)**	**Current**	**Frequency (Hz)**	**Session time**	**Number of sessions**	**Outcome**	**Medications**	**Mean age (years)**	**Female (%)**	**Country**
Kim et al. (21)	Anxiety (≥4/10 on VAS)	RCT	CES (BTCM: made by EM-Tech, Republic of Korea)	25	3	500μA	10 Hz	N/A	21	1.TAI 2.BDI	Nil	25.6 (19–65)	55.56	Korea
			Sham	29								28		
Barclay et al. (15)	Anxiety disorder (DSM-IV)	RCT	CES (Alpha-Stim)	60	5	100 mA	0.5 Hz	60 min	35	1.HAM-A-14 2.HAMD-17	Antidepressants (63.5%)	42.3 (18–65)	67.8	USA
			Sham	55										
Lu (17]	Anxiety disorder (ICD-10)	RCT	CES (Alpha-Stim) + paroxetine	60	6	10–−500 mA	0.5 Hz	60 min	42	HAMA	Paroxetine (100%)	32.6 (18–60)	60	China
			Paroxetine only	60								31.1	66.7	
Chen et al. (16)	Mixed anxiety and depressive disorder (ICD-10)	RCT	CES (Alpha-Stim)	30	3	100–500 μA	0.5 Hz	10~15 min	15	1.SAS 2.SDS	Nil	12 (8–16)	16.7	China
			Sham	30								11	36.7	
Gibson et al. (22)	GAD (STAI≥50)	RCT	CES (Alpha-Stim)	16	nil	50 μA	0.5 Hz	20 min	nil	TAI	Nil	36.64 (22–55)	50	USA
			Sham	16										
Scallet et al. (23)	Chronic hysteria	RCT	CES (Neurotone 101)	5	3	N/A	100Hz	30–40 min	12	1.SRSS–anxiety 2.SRSS–depression 3.SRSS- sleep	allowed	N/A	100	USA
			Sham	5										
Feighner et al. (24)	Suffering from anxiety, depression and insomnia	RCT/ Cross-over	CES (Electrosone 50)	10	2	0.1–0.24 mA	100 Hz	30 min	10	1.Global rating scale–anxiety 2.SDS 3.Global rating scale - sleep	100% on psychotropic agents	42.6 (23–72)	78.9	USA
			Sham	9								39.3		
Rosenthal et al. (25)	Neurotic anxiety and depression	RCT	CES (Russian Electrosone)	11	1	0.1–0.25 mA	100 Hz	30 min	5	1.Anxiety rating scale 2.Depression rating scale 3.Sleep rating scale	minor tranquilizers, tricyclic antidepressants, sleeping medication	43.1 (26–63)	90.90	USA
			Sham	11										

BDI, Beck Depression Inventory; DSM-III-R, Diagnostic and Statistical Manual of Mental Disorders,3th Edition, Revised; DSM-IV, Diagnostic and Statistical Manual of Mental Disorders, 4th Edition; DSM-IV-TR, Diagnostic and Statistical Manual of Mental Disorders, 4th Edition, Text Revision; ESS, Epworth sleepiness Scale; HAM-A Hamilton Anxiety Rating Scale; HAM-A-17, Hamilton Anxiety Rating Scale, 17 itmes; HAMD-17, Hamilton Depression Rating Scale, 17 items; Hz Hertz; K- POMS, Korean version of Profile of Mood States; KSADS, Schedule for Affective Disorders and Schizophrenia—Present and Lifetime Version for Children; mA milliampere; min minutes; POMS, Profile of Mood States; PSQI, Pittsburgh Sleep Quality Index; SAS Zung Self-RatingAnxiety Scale; SDS, Zung Self-Rating Depression Scale; SRSS, National Institutes of Mental Health Self-rating Symptom Scale; TAI, trait anxiety inventory; TD, Tic Disorders; TS, Tourette's disorder; TSGS, Tourette Syndrome Global Scale; μA microampere; VAS, visual analog scales; YGTSS-TTS, Yale Global Tic Severity Scale Total Tic Score.

### 3.2. Risk of bias assessment

**Figure 2:**
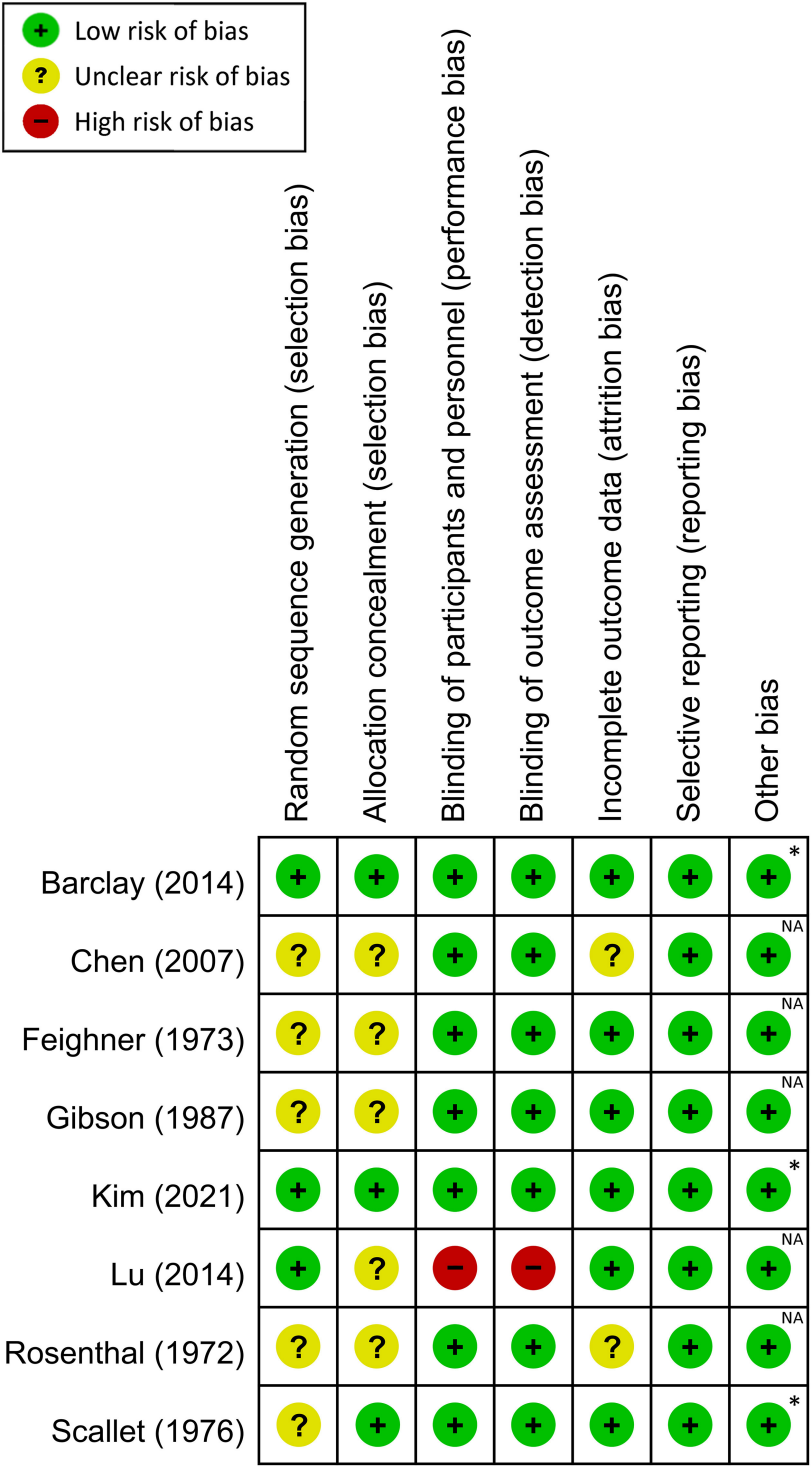
Risk of bias assessment for each study. *Both authors and studies received no financial support from private companies; NA, not available.

A summary of the risks of bias for individual studies included in this meta-analysis is shown in Figure 2 and Supplementary Table 3. Regarding selection bias, the risks for random sequence generation and allocation concealment were deemed unclear in four and six trials, respectively. Performance and detection bias was considered high in one study (17), while the risk of attrition bias was unclear in two trials (16, 25).

### 3.3. Results of syntheses

#### 3.3.1. Primary outcome

**Figure 3:**
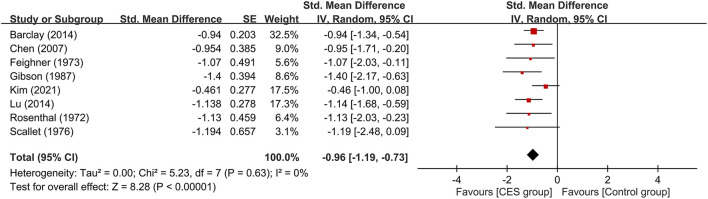
Forest plot comparing the severity of anxiety symptoms between CES and control groups. CES, cranial electrotherapy stimulation; Std, standardized; CI, confidence interval; SE, standard error.

The therapeutic efficacy of CES against anxiety symptoms is shown in Figure 3, which revealed a lower severity of anxiety symptoms in patients receiving CES compared to those without (large treatment effect, SMD: −0.96, 95% CI: −1.19 to −0.73, *p* < 0.00001, 8 studies, 337 participants). There was no heterogeneity on this outcome (I^2^ = 0%).

**Figure 4:**
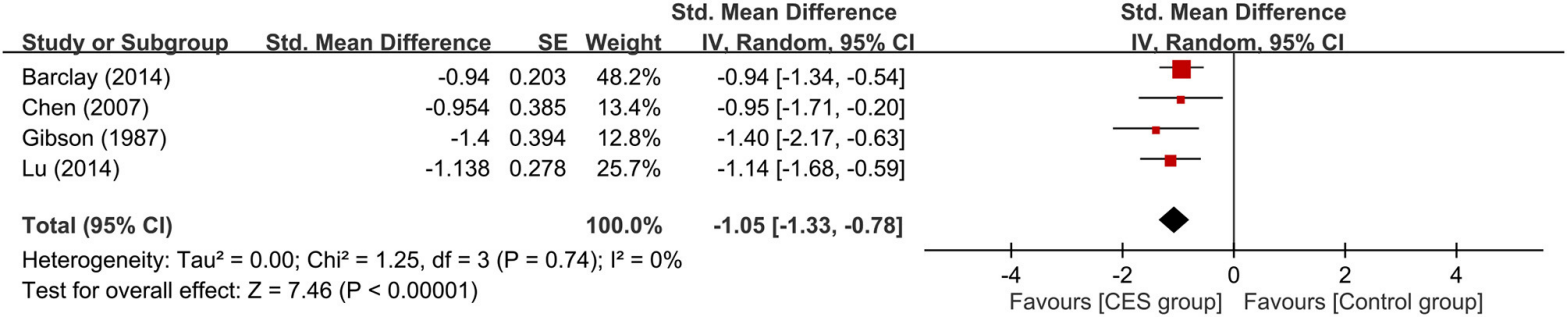
Subgroup analysis of studies that used “Alpha-Stim” for cranial electrotherapy stimulation. CES, cranial electrotherapy stimulation; Std, standardized; CI, confidence interval; SE, standard error.

**Figure 5:**
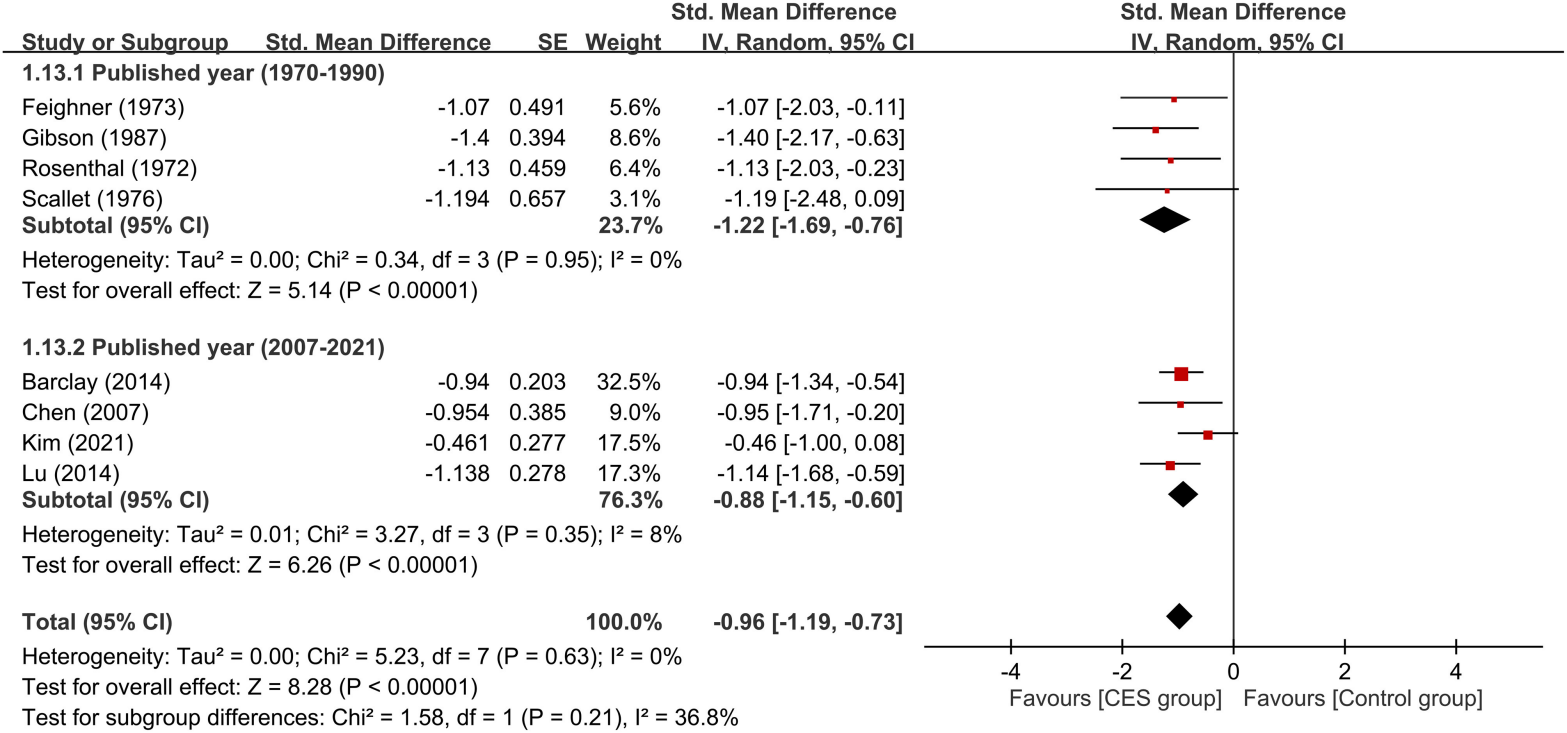
Subgroup analysis on therapeutic efficacy between studies conducted in 1970–1990 and those published in 2007–2021. CES, cranial electrotherapy stimulation; Std, standardized; CI, confidence interval; SE, standard error.

**Figure 6:**
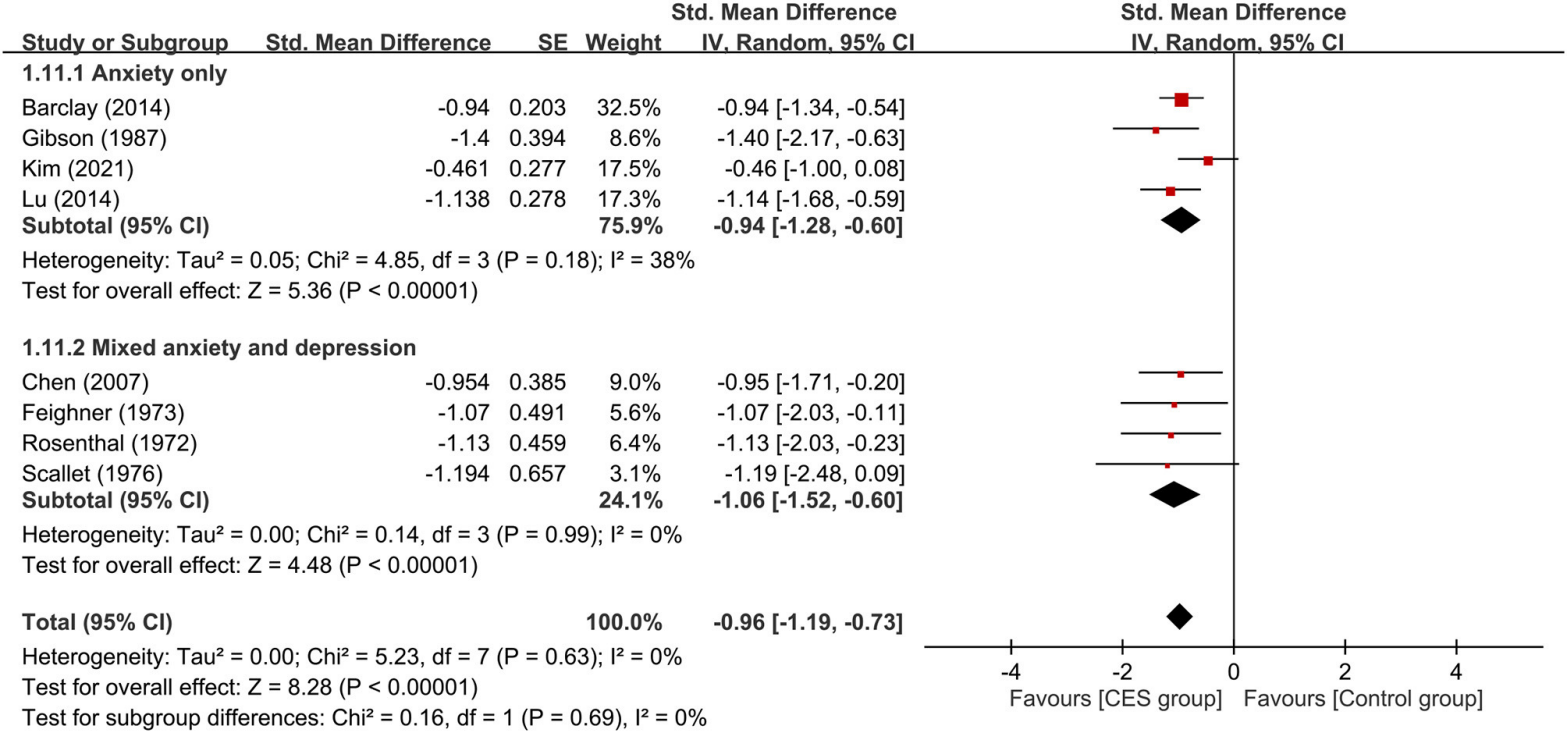
Subgroup analysis of patients with anxiety alone or those with anxiety combined with depressive symptoms. CES, cranial electrotherapy stimulation; Std, standardized; CI, confidence interval; SE, standard error.

**Figure 7:**
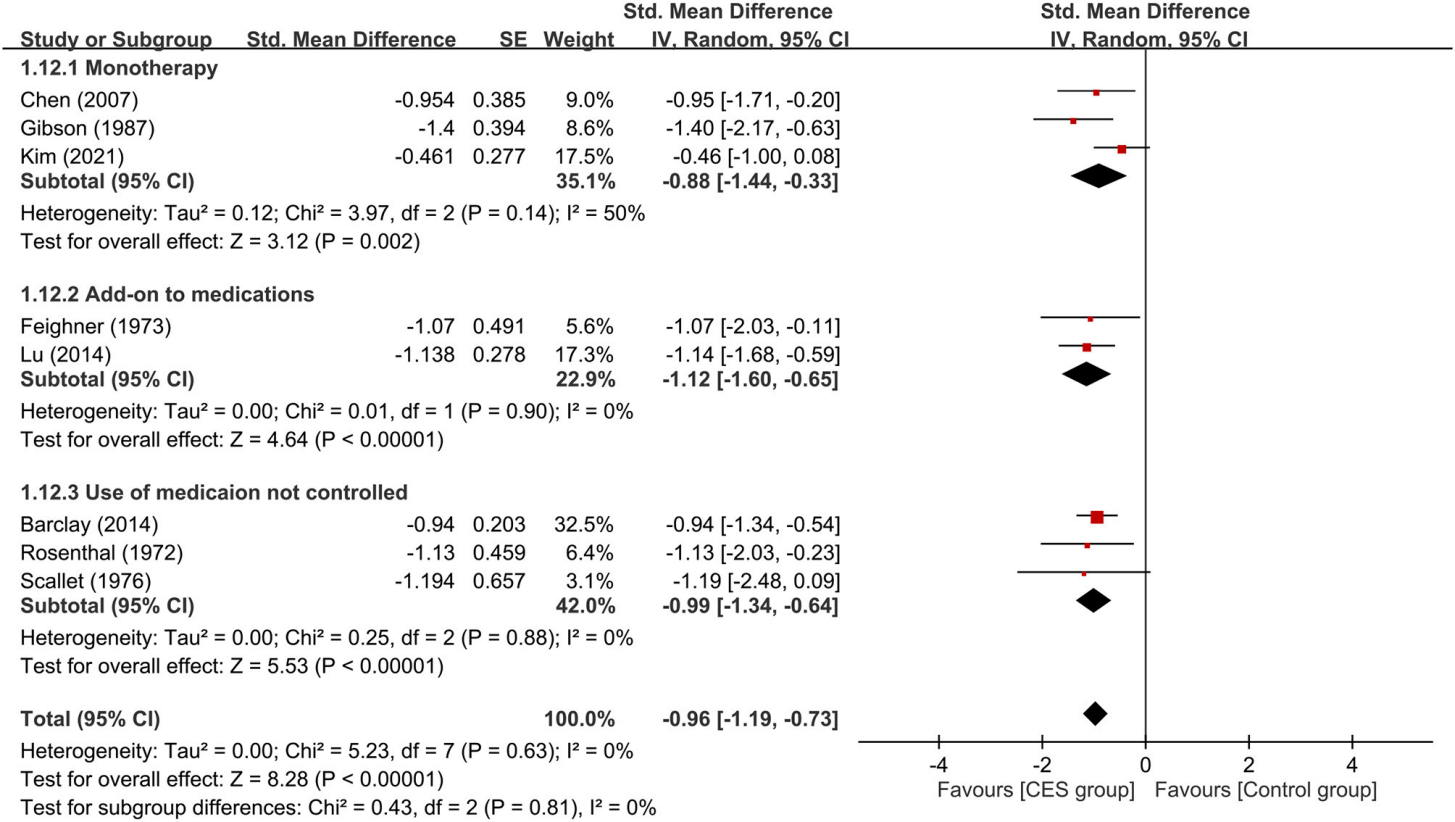
Subgroup analysis of CES as monotherapy vs. add-on therapy. CES, cranial electrotherapy stimulation. CES, cranial electrotherapy stimulation; Std, standardized; CI, confidence interval; SE, standard error.

Subgroup analyses based on the type of CES, anxiety with or without other psychiatric symptoms, CSE as monotherapy or combination therapy are demonstrated in Figures 4–6. Using Alpha-Stim alone for anxiety treatment showed significant anxiety relief in the CES group compared to the control group (large treatment effect, SMD: −1.05, 95% CI: −1.33 to −0.78, *p* < 0.00001, I^2^ = 0%, four studies, 230 participants) (Figure 4). In respect of the period of publication, there was no significant difference in therapeutic efficacy between studies conducted in 1970-1990 and those published in 2007–2021 (Figure 5). Subgroup analyses based on anxiety with or without other psychiatric symptoms (Figure 6) as well as CSE as monotherapy or combination therapy (Figure 7) revealed consistent therapeutic efficacy of CES. The significance of results remained unchanged on sensitivity analysis, suggesting robustness of evidence.

#### 3.3.2. Secondary outcome

**Figure 8:**
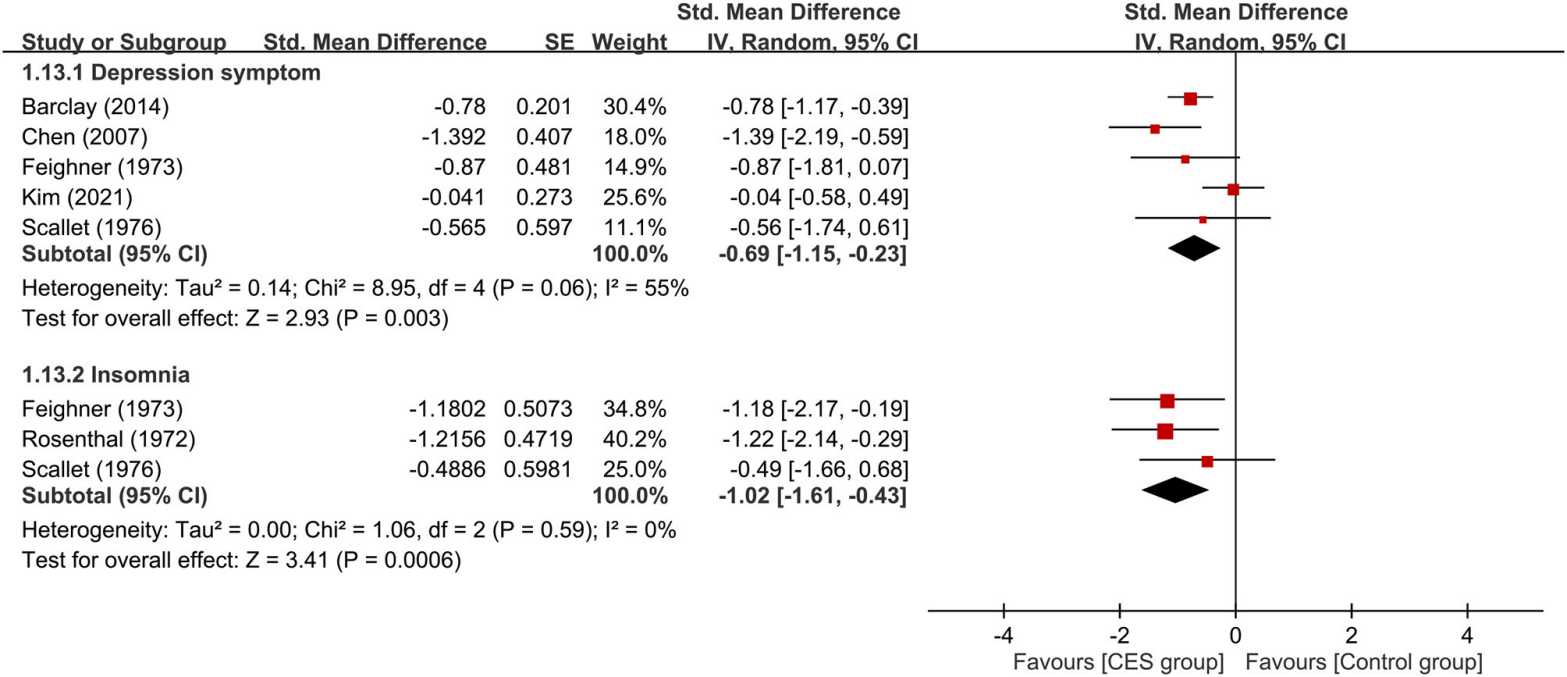
Forest plots comparing the severity of depression symptoms and insomnia between CES and control groups. CES, cranial electrotherapy stimulation; Std, standardized; CI, confidence interval; SE, standard error.

Five studies were available for analyzing the therapeutic efficacy of CES against depression symptoms (15, 16, 21, 23, 24). Merged results showed a lower severity of depression symptoms in patients receiving CES compared to those in the control group (medium treatment effect, SMD: −0.69, 95% CI: −1.15 to −0.23, *p* = 0.003, I^2^ = 55%, five studies, 222 participants) (Figure 8). Analysis of the three studies (23–25) that provided details regarding the effect of CES on insomnia demonstrated a lower severity of insomnia in the CES group than that in the control group (large treatment effect, SMD: −1.02, 95% CI: −1.61 to −0.43, *p* = 0.0006, I^2^ = 0%, three studies, 53 participants) (Figure 8). Regarding acceptability, the use of CES did not increase the risk of treatment–related dropout compared to the control group (RR = 1.26, 95% CI: 0.57 to 2.76, *p* = 0.57, I^2^ = 0%, 324 patients). Sensitivity analysis demonstrated consistent findings on these three outcomes.

#### 3.3.3. Certainty of evidence

Supplementary Table 4 summarizes the overall certainty of evidence on primary and secondary outcomes. The overall certainty of evidence was graded as high in primary outcome (i.e., therapeutic efficacy of CES against anxiety symptoms). For secondary outcomes, the overall certainty of evidence was considered moderate regarding the severity of insomnia and treatment-related dropout rate, while it was deemed low with respect to the severity of depressive symptoms.

## 4. Discussion

To our best knowledge, our meta-analysis is the first to investigate the treatment efficacy of CES that focused particularly on patients experiencing anxiety symptoms. Although there were two previous meta-analyses that examined the treatment efficacy of CES against anxiety symptoms, less than half of the RCTs included in those two studies recruited patients suffering from symptoms of anxiety (18, 19). Moreover, although the most recent meta-analysis conducted subgroup analysis of studies that enrolled patients with anxiety (19), the availability of only three studies may tarnish the quality of evidence (19). In this updated meta-analysis, we excluded RCTs recruiting participants with only physical complaints (e.g., constipation) or those with primary psychiatric diagnoses in which anxiety was not considered an essential part of presenting symptoms (e.g., tic disorder). We further performed subgroup analysis of CES efficacies based on primary diagnoses (i.e., anxiety vs. mixed anxiety and depression), treatment strategies (i.e., monotherapy vs. add-on therapy to medications), and device used for CES (i.e., Alpha-Stim) to investigate their potential influences on the therapeutic efficacy of CES. Furthermore, we not only identified more RCTs (n=8) focusing on patients with anxiety symptoms but also investigated the efficacy of CES against depressive symptoms and insomnia in this population. Our study demonstrated that CES treatment correlated with significantly better improvement in anxiety among patients suffering from symptoms of anxiety than control groups with a large effect size and without significant heterogeneity. Moreover, our secondary analysis showed that CES was more effective than sham control for improving insomnia and depressive symptoms in this population. Our subgroup analyses further found that CES was equally effective regardless of comorbid presentation of depressive symptoms (i.e., a diagnosis of anxiety disorder vs. mixed anxiety and depression) and whether CES was used as monotherapy or add-on therapy to medications. Moreover, subgroup analysis of RCTs using the same device “Alpha-Stim” for CES revealed its efficacy for improving anxiety compared with sham/control groups. We also demonstrated comparable acceptability between CES and sham devices as reflected by a similar dropout rate between the two groups. Overall, our study supported the use of CES for symptoms of anxiety, depression, and insomnia in those experiencing anxiety with fair acceptability and demonstrated the efficacy of “Alpha-Stim”, the most commonly used device for CES, in this patient population.

Although the use of CES dated back as early as the 1960's (11) when it was first approved by the FDA for the treatment of anxiety (29), evidence supporting its treatment efficacy against anxiety symptoms remained limited (11). The first meta-analysis investigating the efficacy of CES for anxiety, which was published in 1995, reported some evidence favoring the use of CES for the treatment of anxiety (18). Nevertheless, most RCTs included in that meta-analysis were of small sample sizes, out-of-date (all before 1986), and recruited patients with other somatic or psychiatric problems (e.g., substance dependence) rather than anxiety symptoms (18). Furthermore, not only are most devices used for CES in that meta-analysis (e.g., Neurotone 101) no longer available (29), but whether different devices had comparable efficacies also remained unclear (29). Although another more recent meta-analysis included more RCTs, more than half of the trials targeted patients presenting with a variety of somatic or psychiatric symptoms other than anxiety and the results showed a significant heterogeneity (19). Moreover, despite an effort to conduct subgroup analysis for participants presenting with symptoms of anxiety, that study (19) only found three RCTs in which one used CES as monotherapy (16), one adopted CES as an add-on treatment to paroxetine (17), and one allowed the use of antidepressants in the majority (63%) of participants (15). Therefore, although that study concluded that CES was effective for improving anxiety in patients suffering from anxiety disorders and perhaps more effective than those experiencing other somatic or psychiatric problems (19), the quality of evidence was limited. Our meta-analysis identified eight trials that targeted patients with a predominant presentation of anxiety demonstrated an ES comparable to that in the previous meta-analysis (ES = −0.96, vs. −1.218, respectively) (19). Despite a similar finding, our results had a better quality of evidence (i.e., rated as high on the GRADE system) without significant heterogeneity to better support the efficacy of CES for relieving symptoms of anxiety in patients with anxiety as their predominant presentation. Our study also showed a larger effect size (ES = −0.96) than that reported in previous meta-analyses that did not particularly target patients with anxiety (ES = −0.537 and ES = −0.625, respectively) (18, 19). Therefore, our study not only provided stronger evidence to support the efficacy of CES for improving anxiety in those with predominant anxiety symptoms but also showed a better efficacy in this patient population compared to those presenting with other somatic or psychiatric symptoms.

With regard to treatment efficacy of CES against depressive symptoms in these patients, we also found that CES was more effective than sham controls. In contrast, a previous meta-analysis, which was unable to provide such information due to a lack of RCTs focusing on patients with predominant anxiety symptoms, only investigated the efficacy of CES against depressive symptoms in those presenting with a variety of somatic or psychiatric symptoms (19). Despite the demonstration of CES efficacy against depressive symptoms in a mixed group of patients in that study, significant heterogeneity was noted (19). On the other hand, there was no such heterogeneity in our results. Our finding, together with the report of no significant heterogeneity in another meta-analysis focusing on participants whose primary complaints were depressive symptoms for which CES was shown to be effective (30), suggested that a mixed patient source may be an important contributing factor to heterogeneity in studies investigating efficacy of CES against depressive or anxiety symptoms. Therefore, targeting participants with similar complaints, rather than recruiting those with mixed medical or psychiatric problems, may be crucial to the study of CES efficacy for specific psychiatric symptoms. Moreover, the smaller ES of efficacy of CES for depression than that for anxiety regardless of the predominant presentation of the patients (i.e., anxiety or depression) implied that CES may be more effective for anxiety than depression.

The finding of our secondary analysis that demonstrated the efficacy of CES against insomnia in patients with predominant anxiety-related manifestations was consistent with that of another meta-analysis focusing on patients with insomnia (31). There was no significant heterogeneity in our study and in that meta-analysis (31), despite the difference in target populations. Again, the selection of participants with similar primary complaints in both studies may highlight the importance of subject homogeneity while investigating the treatment efficacy of CES for psychiatric symptoms.

To identify possible factors that may influence treatment efficacy of CES, we conduct subgroup analyses of participants with different chief complaints (i.e., anxiety only vs. mixed anxiety-depression) and therapeutic strategies of CES (i.e., monotherapy or add-on therapy). Our results demonstrated that neither the predominant symptoms of the participants nor the therapeutic strategies had a significant impact on the efficacy of CES against anxiety. However, the limited number of RCTs in some subgroups (i.e., *n* = 2) from which the results were derived suggested the need for further studies to verify our findings. Nevertheless, our subgroup analysis showed that CES was effective for patients with or without comorbid presentation of depression. Besides, our results supported the use of CES as monotherapy or add-on therapy for anxiety. Moreover, we found comparable acceptability of CES between the study group and the sham controls as reflected by their similar dropout rates. With regard to our subgroup comparison focusing on the period of publication, we did not find significant difference in therapeutic efficacies between devices used in older studies (i.e., 1970–1990) and those reported in more recent investigations (i.e., 2007–2021). Finally, the current study showed that Alpha-Stim (n=4) was an effective device for improving anxiety in this population. Finally, the current study showed that Alpha-Stim (n=4) was an effective device to improve anxiety in this population.

Overall, our study showed that CES was effective for improving not only the symptoms of anxiety, but also depressive symptoms and sleep problems in patients with anxiety. However, it was difficult to determine whether the efficacies of CES for improving sleep and depressive symptoms were related to its anti-anxiety effects. While two meta-analyses also supported the effectiveness of CES for alleviating depressive symptoms and insomnia in patients diagnosed with other psychiatric disorders (e.g. depression and insomnia) (30, 31), a recent RCT demonstrated no significant difference in therapeutic efficacy between CES and sham control for depressive symptoms in patient with major depression (32). Despite our finding of a treatment efficacy of CES against depressive symptoms in patients with anxiety, the ES was much smaller than that against anxiety symptoms. Further studies are required to address the therapeutic effects of CES against different neurotic symptoms.

Despite the inclusion of more RCTs with the selection of participants whose chief complaint was anxiety to avoid heterogeneity, there were several limitations in the current study. First, notwithstanding the large ES for therapeutic efficacy against anxiety in those with predominant anxiety-related symptoms, our results from eight RCTs with 337 participants still need to be confirmed with more large-scale well-controlled clinical investigations. Moreover, the quality of certain studies, especially those published earlier, were down-rated due to unclear description of the randomization process. Nevertheless, given that most studies used sham devices that limited their risks of performance and detection biases, we did not down-grade the certainty of evidence for high risk of bias. Second, although we attempted to identify possible factors (i.e., predominant symptoms and strategies) that may influence the therapeutic efficacy of CES, we were unable to conduct meta-regression for other important factors such as the duration, number of sessions or intensity of stimulation due to the limited numbers of available trials. Third, despite the concern of CES-related side effects, relevant analyses were impossible because most RCTs did not provide such information. Nevertheless, none of the included RCTs reported severe adverse effects. Fourth, although our inclusion of one study that did not use strict diagnostic criteria for anxiety disorders (i.e., self-rate visual analog scales for anxiety > 4 out of 10) may bias our results (21), our leave-one-out sensitivity analysis showed consistent findings after the exclusion of that study. Fifth, because we were unable to assess the long-term effects of CES due to unavailability of such data in our included studies, further clinical trials are needed to investigate the after-effects of CES to design the optimal treatment protocol for patients with anxiety (e.g., the frequency of boost sessions required). Finally, our inclusion of only one study recruiting adolescents (16) limited the extrapolation of our findings on therapeutic efficacy and safety of CES to the younger age groups. Further studies are warranted to address these issues.

## 5. Conclusion

In summary, our study supported the use of CES against the symptoms of anxiety, depression, and insomnia in patients with anxiety as predominant presentation with or without comorbid manifestation of depressive symptoms as monotherapy or an add-on strategy to medications. Our results also showed satisfactory acceptability of CES as well as the efficacy of Alpha-stim. Further clinical trials focusing on those with a chief complaint of anxiety may be crucial for avoiding heterogeneity when investigating the therapeutic efficacy of CES against anxiety symptoms.

## Data availability statement

The original contributions presented in the study are included in the article/Supplementary material, further inquiries can be directed to the corresponding author.

## Author contributions

Conceptualization: F-CC, C-KS, YC, and Y-SC. Data curation: WC, R-FT, and H-JC. Methodology: M-YW and Y-CC. Supervision: F-CC, C-KS, YC, Y-SC, and K-CH. Writing—original draft: F-CC, C-KS, Y-SC, and K-CH. Writing—review and editing: Y-CC and K-CH. All authors contributed to the article and approved the submitted version.
