# Supplementary material for: Efficacy of electrical cranial stimulation for treatment of psychiatric symptoms in patients with anxiety: A systematic review and meta-analysis
Source: Front Psychiatry. 2023 Apr 6;14:1157473. doi: 10.3389/fpsyt.2023.1157473 (PMC10115990; doi:10.3389/fpsyt.2023.1157473)
Supplement: Supplementary file 1 [file Table_1.docx]

**Supplemental Table 1.** Search strategies for Medline

| Database | # | Search syntax |
| --- | --- | --- |
| **MEDLINE (Ovid)** | 1 | ("Cranial Electrotherapy Stimulation" OR "Cranial Electrical Stimulation" OR "CES" OR "Electric Stimulation Therapy").mp |
|  | 2 | ("Anxiety" OR "Anxiousness" OR "Anxiety disorders").mp |
|  | 3 | exp "anxiety"/ |
|  | 4 | 1 AND (2 OR 3) |
|  | 5 | 4 AND (randomized controlled trial.pt. or controlled clinical trial.pt. or randomi*ed.ab. or placebo.ab. or drug therapy.fs. or randomly.ab. or trial.ab. or groups.ab. not (exp animals/ not humans.sh.)) |

**Supplemental Table 2** Reasons for study exclusion

| **Reason** | **Number of excluded studies** | | **References** | |
| --- | --- | --- | --- | --- |
| No outcome for anxiety symptoms | 14 | [1-14] | |  |
| Not for patients with anxiety | 4 | [15-18] | |  |
| No analyzable data | 1 | [19] | |  |
| Not RCT | 4 | [20-23] | |  |
| Not CES | 4 | [24-27] | |  |

CES cranial electrical stimulation

RCT randomized controlled trials

**References**

1. McClure, D., et al., A Pilot Study of Safety and Efficacy of Cranial Electrotherapy Stimulation in Treatment of Bipolar II Depression. J Nerv Ment Dis, 2015. 203(11): p. 827-35.

2. Mischoulon, D., et al., Efficacy and safety of a form of cranial electrical stimulation (CES) as an add-on intervention for treatment-resistant major depressive disorder: A three week double blind pilot study. J Psychiatr Res, 2015. 70: p. 98-105.

3. Lu, T., et al., Percutaneous mastoid electrical stimulator improves Poststroke depression and cognitive function in patients with Ischaemic stroke: a prospective, randomized, double-blind, and sham-controlled study. BMC Neurol, 2020. 20(1): p. 217.

4. Wagenseil, B., et al., The effect of cranial electrotherapy stimulation on sleep in healthy women. Physiol Meas, 2018. 39(11): p. 114007.

5. Rintala, D.H., et al., Feasibility of using cranial electrotherapy stimulation for pain in persons with Parkinson's disease. Parkinsons Dis, 2010. 2010: p. 569154.

6. Weiss, M.F., The treatment of insomnia through the use of electrosleep: an EEG study. J Nerv Ment Dis, 1973. 157(2): p. 108-20.

7. Taylor, A.G., et al., Cranial electrical stimulation improves symptoms and functional status in individuals with fibromyalgia. Pain Manag Nurs, 2013. 14(4): p. 327-335.

8. Lawson, D., et al., Efficacy of microcurrent therapy for treatment of acute knee pain: A randomized double-blinded controlled clinical trial. Clinical Rehabilitation, 2020. 35(3): p. 390-398.

9. Gabis, L., B. Shklar, and D. Geva, Immediate influence of transcranial electrostimulation on pain and beta-endorphin blood levels: an active placebo-controlled study. Am J Phys Med Rehabil, 2003. 82(2): p. 81-5.

10. Gabis, L., et al., Pain reduction using transcranial electrostimulation: a double blind "active placebo" controlled trial. J Rehabil Med, 2009. 41(4): p. 256-61.

11. Katsnelson, Y., et al., Temporary pain relief using transcranial electrotherapy stimulation: results of a randomized, double-blind pilot study. Conf Proc IEEE Eng Med Biol Soc, 2004. 2004: p. 4087-90.

12. Lichtbroun, A.S., M.M. Raicer, and R.B. Smith, The treatment of fibromyalgia with cranial electrotherapy stimulation. J Clin Rheumatol, 2001. 7(2): p. 72-8; discussion 78.

13. Tan, G., et al., Using cranial electrotherapy stimulation to treat pain associated with spinal cord injury. J Rehabil Res Dev, 2006. 43(4): p. 461-74.

14. Patel, S., et al., A randomised controlled trial investigating the clinical and cost-effectiveness of Alpha-Stim AID cranial electrotherapy stimulation (CES) in patients seeking treatment for moderate severity depression in primary care (Alpha-Stim-D Trial). Trials, 2022. 23(1): p. 250.

15. Roh, H.T. and W.Y. So, Cranial electrotherapy stimulation affects mood state but not levels of peripheral neurotrophic factors or hypothalamic- pituitary-adrenal axis regulation. Technol Health Care, 2017. 25(3): p. 403-412.

16. Chang, W.D., et al., Cranial Electrotherapy Stimulation to Improve the Physiology and Psychology Response, Response-Ability, and Sleep Efficiency in Athletes with Poor Sleep Quality. Int J Environ Res Public Health, 2022. 19(4).

17. Wu, W.J., et al., A double-blind, randomized, sham-controlled study of cranial electrotherapy stimulation as an add-on treatment for tic disorders in children and adolescents. Asian J Psychiatr, 2020. 51: p. 101992.

18. Tan, G., et al., Efficacy of cranial electrotherapy stimulation for neuropathic pain following spinal cord injury: a multi-site randomized controlled trial with a secondary 6-month open-label phase. J Spinal Cord Med, 2011. 34(3): p. 285-96.

19. Moore, J.A., et al., A double-blind study of electrosleep for anxiety and insomnia. Biol Psychiatry, 1975. 10(1): p. 59-63.

20. Shekelle, P.G., et al., Benefits and Harms of Cranial Electrical Stimulation for Chronic Painful Conditions, Depression, Anxiety, and Insomnia: A Systematic Review. Ann Intern Med, 2018. 168(6): p. 414-421.

21. Ching, P.Y., et al., Efficacy and Tolerability of Cranial Electrotherapy Stimulation in the Treatment of Anxiety: A Systemic Review and Meta-Analysis. Front Psychiatry, 2022. 13: p. 899040.

22. Padjen, A.L., M. Dongier, and T. Malec, Effects of cerebral electrical stimulation on alcoholism: a pilot study. Alcohol Clin Exp Res, 1995. 19(4): p. 1004-10.

23. Mannu, P., et al., Radio electric treatment vs. Es-Citalopram in the treatment of panic disorders associated with major depression: an open-label, naturalistic study. Acupunct Electrother Res, 2009. 34(3-4): p. 135-49.

24. Luedtke, K., et al., Effectiveness of anodal transcranial direct current stimulation in patients with chronic low back pain: design, method and protocol for a randomised controlled trial. BMC Musculoskelet Disord, 2011. 12: p. 290.

25. Cha, Y.H., D. Urbano, and N. Pariseau, Randomized Single Blind Sham Controlled Trial of Adjunctive Home-Based tDCS after rTMS for Mal De Debarquement Syndrome: Safety, Efficacy, and Participant Satisfaction Assessment. Brain Stimul, 2016. 9(4): p. 537-44.

26. Taylor, D.N., et al., The effects of cranial TENS on measures of autonomic somatic and cognitive activity. Acupunct Electrother Res, 1989. 14(1): p. 29-42.

27. Taylor, D.N., C.T. Lee, and J.J. Katims, Effects of cranial transcutaneous electrical nerve stimulation in normal subjects at rest and during psychological stress. Acupunct Electrother Res, 1991. 16(1-2): p. 65-74.

| **Supplemental Table 3.** Risk of bias assessment   1. Lu (2014)  \| Categories \| Risk \| Reasons \| \| --- \| --- \| --- \| \| Random sequence \| Low \| Randomization by using random number table \| \| Allocation concealment \| Unknown \| Allocation concealment process unclear \| \| Performance bias \| High \| No use of sham device and participant aware of their treatment \| \| Detection bias \| High \| No use of sham device and participant aware of their treatment (self-rating scale) \| \| Attrition bias \| Low \| Little missing data \| \| Reporting bias \| Low \| Outcomes analyzed in accordance with a pre-specified plan \| \| Other bias \| Low \| No other obvious risk of bias \|  1. Chen (2007)  \| Categories \| Risk \| Reasons \| \| --- \| --- \| --- \| \| Random sequence \| Unknown \| Randomization process unclear \| \| Allocation concealment \| Unknown \| Allocation concealment process unclear \| \| Performance bias \| Low \| Use of sham device and allocation blinded to participants \| \| Detection bias \| Low \| All participants and assessors were blinded from allocation of treatment \| \| Attrition bias \| Unknown \| No information about missing data \| \| Reporting bias \| Low \| Outcomes analyzed in accordance with a pre-specified plan \| \| Other bias \| Low \| No other obvious risk of bias \|  1. Gibson (1987)  \| Categories \| Risk \| Reasons \| \| --- \| --- \| --- \| \| Random sequence \| Unknown \| Randomization process unclear \| \| Allocation concealment \| Unknown \| Allocation concealment process unclear \| \| Performance bias \| Low \| Use of sham device and allocation blinded to participants \| \| Detection bias \| Low \| All participants and assessors were blinded from allocation of treatment \| \| Attrition bias \| Low \| Little missing data \| \| Reporting bias \| Low \| Outcomes analyzed in accordance with a pre-specified plan \| \| Other bias \| Low \| No other obvious risk of bias \|  1. Scallet (1976)  \| Categories \| Risk \| Reasons \| \| --- \| --- \| --- \| \| Random sequence \| Unknown \| Randomization process unclear \| \| Allocation concealment \| Low \| Allocation by person not involved in evaluation and treatment \| \| Performance bias \| Low \| Use of sham device and allocation blinded to participants \| \| Detection bias \| Low \| All participants and assessors were blinded from allocation of treatment \| \| Attrition bias \| Low \| Little missing data \| \| Reporting bias \| Low \| Outcomes analyzed in accordance with a pre-specified plan \| \| Other bias \| Low \| No other obvious risk of bias \|  1. Feighner (1973)  \| Categories \| Risk \| Reasons \| \| --- \| --- \| --- \| \| Random sequence \| Unknown \| Randomization process unclear \| \| Allocation concealment \| Unknown \| Allocation concealment process unclear \| \| Performance bias \| Low \| Use of sham device and allocation blinded to participants \| \| Detection bias \| Low \| All participants and assessors were blinded from allocation of treatment \| \| Attrition bias \| Low \| Little missing data \| \| Reporting bias \| Low \| Outcomes analyzed in accordance with a pre-specified plan \| \| Other bias \| Low \| No other obvious risk of bias \|  1. Rosenthal (1972)  \| Categories \| Risk \| Reasons \| \| --- \| --- \| --- \| \| Random sequence \| Unknown \| Randomization process unclear \| \| Allocation concealment \| Unknown \| Allocation concealment process unclear \| \| Performance bias \| Low \| Use of sham device and allocation blinded to participants \| \| Detection bias \| Low \| All participants and assessors were blinded from allocation of treatment \| \| Attrition bias \| Unknown \| No information about missing data \| \| Reporting bias \| Low \| Outcomes analyzed in accordance with a pre-specified plan \| \| Other bias \| Low \| No other obvious risk of bias \|   **Supplemental Table 4.** Summary of findings for the main comparison | | | | | | |
| --- | --- | --- | --- | --- | --- | --- | --- | --- | --- | --- | --- | --- | --- | --- | --- | --- | --- | --- | --- | --- | --- | --- | --- | --- | --- | --- | --- | --- | --- | --- | --- | --- | --- | --- | --- | --- | --- | --- | --- | --- | --- | --- | --- | --- | --- | --- | --- | --- | --- | --- | --- | --- | --- | --- | --- | --- | --- | --- | --- | --- | --- | --- | --- | --- | --- | --- | --- | --- | --- | --- | --- | --- | --- | --- | --- | --- | --- | --- | --- | --- | --- | --- | --- | --- | --- | --- | --- | --- | --- | --- | --- | --- | --- | --- | --- | --- | --- | --- | --- | --- | --- | --- | --- | --- | --- | --- | --- | --- | --- | --- | --- | --- | --- | --- | --- | --- | --- | --- | --- | --- | --- | --- | --- | --- | --- | --- | --- | --- | --- | --- | --- | --- | --- | --- | --- | --- | --- | --- | --- | --- | --- | --- | --- | --- | --- | --- | --- | --- | --- | --- |
| Outcomes | Effect (Risk) | | Relative effect (95% CI) | № of participants  (studies) | Certainty of the evidence (GRADE) | Comments |
|  | Intervention group | Control group |  |  |  |  |
| Therapeutic efficacy of CES on anxiety symptoms | - | - | SMD -0.96 (-1.19 to -0.73) | 337  (8 studies) | ⨁⨁⨁⨁ High | - |
| Therapeutic efficacy of CES on severity of depression symptoms | - | - | SMD -0.69  (-1.15 to -0.23) | 222 (5 studies) | ⨁⨁◯◯ Low | a, b |
| Therapeutic efficacy of CES on severity of insomnia | - | - | SMD -1.02 (-1.61 to -0.43) | 53 (3 studies) | ⨁⨁⨁◯ Moderate | a |
| Treatment-related drop-out rate | 13/165 | 10/159 | OR 1.26  (0.57 to 2.76) | 324  (4 studies) | ⨁⨁⨁◯ Moderate | a |

**Comments:**

^a^wide 95% confidence interval

^b^The I square is more than 50%.

**GRADE Working Group grades of evidence:**
-**High certainty**: We are very confident that the true effect lies close to that of the estimate of the effect
-**Moderate certainty**: We are moderately confident in the effect estimate: The true effect is likely to be close to the estimate of the effect, but there is a possibility that it is substantially different.
-**Low certainty**: Our confidence in the effect estimate is limited: The true effect may be substantially different from the estimate of the effect.
-**Very low certainty**: We have very little confidence in the effect estimate: The true effect is likely to be substantially different from the estimate of effect.
